# In Vivo Activity of LCB 01-0699, a Prodrug of LCB 01-0648, against *Staphylococcus aureus*

**DOI:** 10.3390/molecules22122096

**Published:** 2017-11-29

**Authors:** Sang-Hun Oh, Hee-Soo Park, Jun-Hyung Lee, Sung-Yun Baek, Sang-Eun Chae, Kyuman Oh, Young Lag Cho, Jin-Hwan Kwak

**Affiliations:** 1 School of Life Science, Handong Global University, Pohang 37554, Korea; osh8755@naver.com (S.-H.O.); gohyo9@gmail.com (J.-H.L.); 2 Institute of Agricultural Science & Technology, School of Food Science and Biotechnology, Kyungpook National University, Daegu 41566, Korea; phsoo97@knu.ac.kr; 3 LegoChem BioSci. Inc., Daejeon 34302, Korea; bsy@legochembio.com (S.-Y.B.); sangeun@legochembio.com (S.-E.C.); kyuman@legochembio.com (K.O.); young@legochembio.com (Y.L.C.)

**Keywords:** LCB01-0699, LCB01-0648, oxazolidinone, in vivo activity, skin infection

## Abstract

**LCB01-0648** is a novel oxazolidinone compound that shows potent antibacterial activities against most Gram-positive cocci, including the multi-drug resistant *Staphylococcus aureus*. In this study, in vivo activity of **LCB01-0699**, a **LCB01-0648** prodrug, against *S. aureus* was evaluated in comparison with that of **Linezolid**. The results of the systemic infection study demonstrated that **LCB01-0699** was more potent than **Linezolid** against methicillin-susceptible and -resistant *S. aureus* strains. The in vivo efficacy of **LCB01-0699** against methicillin-susceptible and -resistant *S. aureus* strains in a skin infection model showed more potent activity than **Linezolid**. **LCB01-0699** shows potent in vivo activity against methicillin-susceptible and -resistant *S. aureus* strains, suggesting that **LCB01-0699** would be a novel candidate for the treatment of these infectious diseases caused by *S. aureus*.

## 1. Introduction

Multidrug-resistant Gram-positive bacteria, including methicillin-resistant *Staphylococcus aureus* (MRSA), beta-lactam-resistant *Streptococcus pneumoniae*, and vancomycin-resistant enterococci (VRE), are widespread around the world, and are a major concern for nosocomial infections with high morbidity and mortality [1,2]. Although a rapid increase in resistant bacteria is occurring, the development of novel antibiotics for use in the treatment of infectious diseases is limited [3]. Therefore, developing novel antibiotics for treatment of these infectious diseases caused by multidrug resistant Gram-positive bacteria is necessary [4,5].

Oxazolidinones are a new class of synthetic antibiotics that bind to 23S ribosomal RNA, a component of the 50S subunit of the bacterial ribosome, thereby inhibiting the initiation of protein synthesis [6,7]. Because oxazolidinones have potent antimicrobial activities against Gram-positive bacteria, oxazolidinones have been used for the treatment of soft- and skin-tissue infectious diseases caused by multidrug-resistant Gram positive cocci [8,9]. **Linezolid** is the first oxazolidinone antibiotic approved by the United States Food and Drug Administration (USFDA) in 2000 [10]. While **Linezolid** has been used for infectious diseases, several **Linezolid**-resistant staphylococci have been reported globally [11]. To overcome **Linezolid**-resistant *S. aureus* (LRSA), development of second-generation oxazolidinones with potent activities against **Linezolid**-resistant *S. aureus* or other multi-drug resistant Gram-positive cocci is required. Recently, Sivextro (**tedizolid phosphate**), a second-generation oxazolidinone, was approved by the USFDA for the treatment of acute bacterial skin and skin structure infections (ABSSSI) caused by certain Gram-positive bacteria [12].

**Figure 1:**
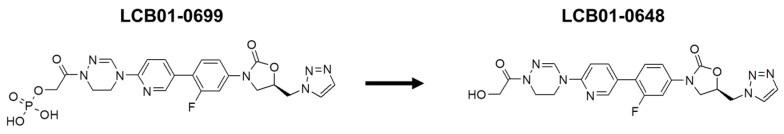
Chemical structures of **LCB01-0648** and **LCB01-0699**.

Previously, we reported on the antibacterial activities of a novel oxazolidinones **LCB01-0648**, containing cyclic amidrazone [13]. **LCB01-0648** showed potent antibacterial activities against clinically isolated Gram-positive cocci, suggesting that **LCB01-0648** could be a good antibiotic candidate for treatment of infectious diseases caused by Gram-positive cocci [13]. While **LCB01-0648** has potent antibacterial activities against multi-drug Gram-positive cocci in vitro, the in vivo activity of **LCB01-0648** had not yet been examined. To examine the in vivo activity of **LCB01-0648**, **LCB01-0699**, a prodrug of **LCB01-0648** was used (Figure 1). First, pharmacokinetic results demonstrated that most **LCB01-0699** converts to the active form **LCB01-0648** in the rat model. Two infection models, including systemic- and skin-infection models, were used for examination of the in vivo efficacy of **LCB01-0699**, and found that **LCB01-0699** was similarly active to or more active than **Linezolid** against methicillin-susceptible and -resistant *S. aureus*.

## 2. Results

**Figure 2:**
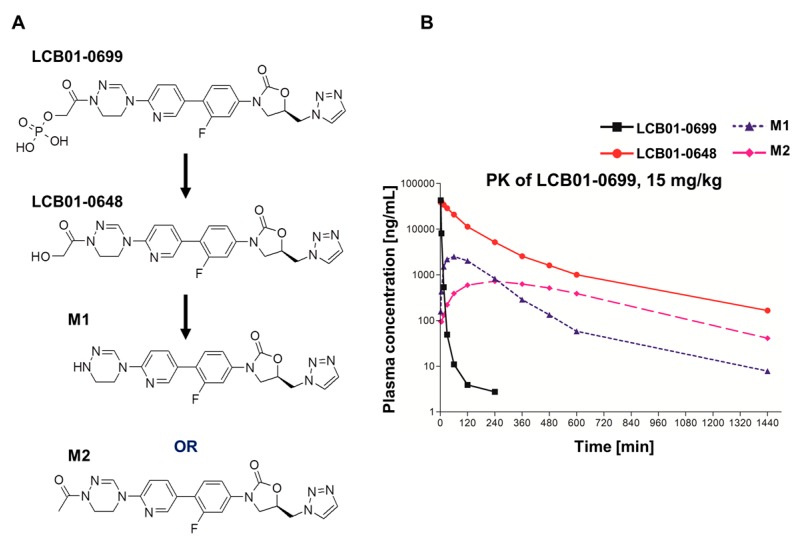
(**A**) Proposed biotransformation of **LCB01-0699**; (**B**) Average (±SD) plasma concentration of **LCB01-0699** and their metabolites versus time plots of 24 subjects after administration of 15 mg/kg **LCB01-0699**.

Previously, we reported that **LCB01-0648** had potent in vitro activity and minimal safety issues [13]. To examine whether **LCB01-0699** was really converted to **LCB01-0648** in vivo, a pharmacokinetic study was conducted in a rat model system. A single dose of **LCB01-0699** was administrated as a bolus injection through the tail vein, and serum samples were analyzed by high-performance liquid chromatography (HPLC) assay. As shown in Figure 2, most of the **LCB01-0699** was converted to **LCB01-0648** rapidly. The C_max_ (peak concentration), T_max_ (time required to reach maximum) and AUC_last_ (area under the concentration-time curve from time zero to the last sampling time) of **LCB01-0699** were found to be 42.9 mg L^−1^, 0.03 h and 5.08 mg·h L^−1^, respectively. The C_max_, T_max_ and AUC_last_ of **LCB01-0648** were found to be 41.1 mg L^−1^, 0.07 h and 85.0 mg·h L^−1^, respectively (Table 1). These results demonstrate that most of the prodrug **LCB01-0699** converted to **LCB01-0648**.

To examine the in vivo activities of **LCB01-0699**, we first used a systemic-infection mouse model. The median effective dose needed to protect 50% of the mice (ED_50_) of **LCB01-0699** were 6.20 and 2.23 mg/kg of body weight against *S. aureus* Giorgio (methicillin-susceptible *S. aureus*) and *S. aureus* P125 (methicillin-resistant *S. aureus*), respectively, when orally administered (p.o.) (Table 2). Against infection caused by methicillin-susceptible and -resistant *S. aureus*, The median effective dose needed to protect 50% of the mice (ED_50s_) of **LCB01-0699** were 5.51 and 2.73 mg/kg of body weight, respectively, when **LCB01-0699** was administered by the subcutaneous route (s.c.). These results demonstrate that **LCB01-0699** is more potent than **Linezolid** against methicillin-susceptible and -resistant *S. aureus*.

**Figure 3:**
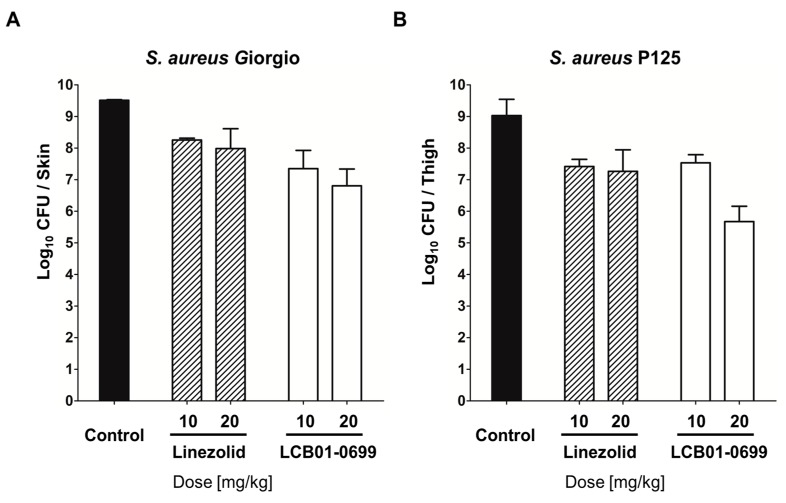
In vivo activities of **LCB01-0699** and **Linezolid** against (**A**) *S. aureus* Giorgio (methicillin-susceptible *S. aureus*) and (**B**) *S. aureus* P125 (methicillin-resistant *S. aureus*) in mouse model of skin infection. Each bar represents mean ± SD. CFU: colony-forming units.

We then examined the in vivo effect of **LCB01-0699** by using a soft-tissue infection model caused by *S. aureus* Giorgio (methicillin-susceptible *S. aureus*) and *S. aureus* P125 (methicillin-resistant *S. aureus*). As shown Figure 3, **LCB01-0699** showed reduced bacterial count in a dose-dependent manner in air-pouch fluid. At a concentration of 20 mg/kg of body weight, the antibacterial activity of **LCB01-0699** was better than that of **Linezolid** in both methicillin-susceptible and -resistant *S. aureus* strains, suggesting that **LCB01-0699** has potent activity in soft-tissue infection model.

**Figure 4:**
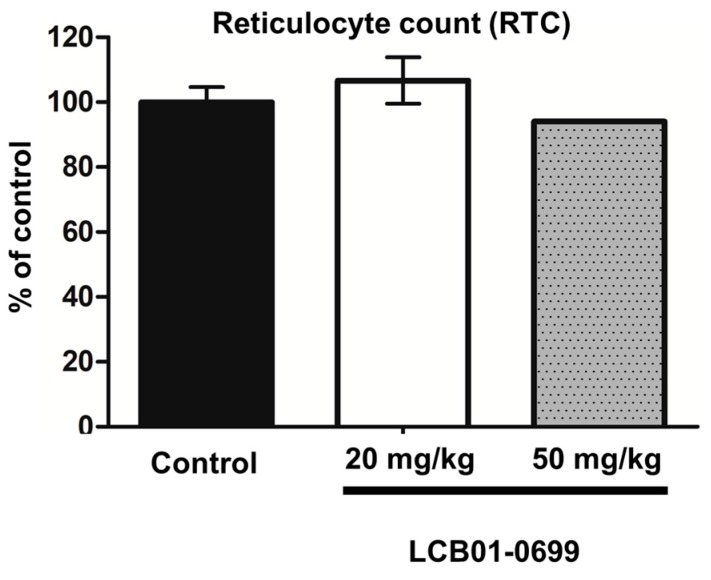
Myelosuppression toxicity of **LCB01-0699**. Each bar represents mean ± SD.

Because myelosuppression is associated with **Linezolid** [14], we conducted a myelosuppression assay. Similar to **LCB01-0648**, **LCB01-0699** was not able to cause any change of reticulocyte count (RTC, %) (Figure 4), suggesting that **LCB01-0699** may not be associated with myelosuppression.

## 3. Discussion

Oxazolidinones are a novel group of antibiotics that have potent activities against Gram-positive pathogens [8]. Until now, two agents, **Linezolid** and tedizolid, have been approved in USFDA. **Tedizolid phosphate** (TR-701), a second-generation oxazolidinone, was developed for the treatment of skin-infection diseases caused by Gram-positive cocci including *S. aureus* [12]. Unlike **Linezolid**, **Tedizolid** uses a prodrug form—**Tedizolid phosphate**—for treatment, and this prodrug is rapidly converted by phosphatases to tedizolid in humans.

Previously, we demonstrated that **LCB01-0648**, a novel oxazolidinone agent, has potent in vitro antibacterial activities of **LCB01-0648** against Gram-positive cocci [15]. In this study, we further examined the in vivo activity of **LCB01-0699**, the prodrug form of **LCB01-0648**. **LCB01-0699**, the **LCB01-0648** phosphate form, is quickly converted to the active form **LCB01-0648** in rat plasma after intravenous injection. **LCB01-0699** was more active than **Linezolid** against both methicillin-susceptible and -resistant *S. aureus* strains in systemic and skin mouse models, suggesting that **LCB01-0699** could be a good oxazolidinone agent for treatment of skin infection caused by methicillin-resistant *S. aureus*. Because **LCB01-0699** showed less bone marrow toxicity (Figure 4), **LCB01-0699** is considered a potential antibacterial candidate, with high activities and low toxicity. While our previous study provided in vitro activities of **LCB01-0648** against **Linezolid**-resistant *S. aureus* or other Gram-positive cocci [13], we did not provide in vivo activity of **LCB01-0699** against **Linezolid**-resistant *S. aureus* or other Gram-positive cocci. Therefore, further in vivo analyses should be carried out to better understand the antibacterial activity of **LCB01-0699** against multi-drug resistant Gram-positive cocci, including **Linezolid**-resistant *S. aureus*.

## 4. Materials and Methods

### 4.1. Antimicrobial Agents and Bacterial Strains

**LCB01-0699** and **Linezolid** were synthesized at the LegoChem Bioscience, Inc., Daejeon, Korea. For in vivo experiments, methicillin-susceptible *S. aureus* Giorgio, obtained from LG Chem, Ltd., Seoul, Korea, and methicillin-resistant *S. aureus* strain P125 were previously selected through the screening of clinical isolates in the previous study [16].

### 4.2. Systemic Infection Model

*S. aureus* Giorgio or P125 were cultured in Mueller-Hinton agar (MHA, Difco, Sparks, MD, USA) medium at 37 °C for 18 h and were suspended in 5% gastric mucin (Sigma-Aldrich Co., St. Louis, MO, USA). Four-week-old male mice weighing 19 to 21 g, with each group being 5 mice in a single cage, were injected intraperitoneally with the bacterial suspension corresponding to an inoculum range of 5 to 10 times the minimum lethal dose (MLD) of bacteria. Four dose levels were used for each antibiotic, depending on the Minimum inhibitory concentration (MIC) of the compound. **LCB01-0699** and **Linezolid** at various dose regimens were administered orally (p.o.) or subcutaneously (s.c.), twice at 1 and 4 h post infection. Mortality was recorded for 7 days, and the median effective dose needed to protect 50% of the mice (ED_50_) was calculated by the Probit method.

### 4.3. Soft Tissue Infection Model

Four-week-old female IcrTacSam (ICR) mice (18 to 23 g) were used in groups of three for each dose. After 24 h, challenge bacterial strains with 5% mucin (Sigma-Aldrich Co., St. Louis, MO, USA) were infected into the air pouches. **LCB01-0699** and **Linezolid** were orally administered (p.o.) at 0 h after the bacterial infection. After 24 h, 3 mL of saline was injected into the pouch, and the fluid from the pouch was removed immediately. The fluid from the pouch was serial diluted with saline and were then plated on Mueller-Hinton agar plates to count the number of residual bacteria.

### 4.4. Mice and Ethics Statement

For in vivo experiments, male or female IcrTacSam (ICR) mice were purchased from the Daehan Bio Link Co., Ltd., Eumseong, Korea. All animals were maintained in the specific-pathogen-free (SPF) facility, where the cages in the animal room were maintained at 22 ± 2 °C temperature and 55 ± 1.0% relative humidity. The facilities were adjusted day and night at 12-h intervals (9 a.m. and 9 p.m.). Before the experiment, the mice were separated into cages by weight. All animal experiments were conducted in accordance with the ethical guidelines of the Ethics Review Committee for Animal Experimentation at Handong Global University (Pohang, Korea) (protocol #HGU-2010-04 and #HGU-20151022-003).

### 4.5. Pharmacokinetic Analysis

A total of 3 male Sprague Dawley (SD) rats (Orient Bio Inc., Seongnam, Korea weighing 280~311 g) were used in this study. Animals were fasted overnight before and for 4 h after dosing. The animal room was controlled for illumination (12 h light/dark cycle), temperature (19~25 °C) and relative humidity (>40%). The phosphate prodrug **LCB01-0699** (7.5 mg/mL) was administered as an intravenous bolus dose at 15 mg/kg via tail vein. Following intravenous administration, blood samples (0.5 mL) were collected from the jugular vein prior to dosing, and at 0.033, 0.083, 0.25, 0.5, 1, 2, 4, 6, 8, 10 and 24 h post-dose. Blood samples were collected in tubes containing sodium-heparin, and centrifuged at 14,000 rpm for 5 min at 4 °C to separate plasma from the blood samples. Following centrifugation, 100 μL aliquots of plasma were stored at −70 °C until high-performance liquid chromatography with tandem mass spectrometry (HPLC/MS/MS) analysis. The plasma concentrations of **LCB01-0699**, **LCB01-0648** and metabolites (**M1**, **M2**) were determined by protein precipitation, and by using the high-performance liquid chromatography with tandem mass spectrometry (HPLC/MS/MS) method. The pharmacokinetic parameters were determined for **LCB01-0648** and metabolites from individual plasma concentration-time data using the linear log trapezoidal calculation method. A non-compartmental analysis of Phoenix WinNonlin^®^ Professional 6.4 (Certara USA, Inc., Princeton, NJ, USA) was used to calculate parameters.

### 4.6. Myelosuppression Assay

A myelosuppression assay was carried out as previously described [13]. Briefly, female C3H/HeJ (C3H) mice (6~7 weeks) were purchased from Orient Bio. Charles River Laboratories (Seongnam, Korea). **LCB01-0699** (20 or 50 mg/kg) was orally administered (p.o.) once daily for 4 days using a sonde attached to disposable syringes. Mice were sacrificed by ether 4 days after treatment. All animals had been fasted for approximately 16 h prior to sacrifice. Blood samples were collected from the vena cava and were put into a tube containing EDTA (Ethylenediaminetetraacetic acid) (Becton Dickinson, Sparks, MD, USA). Samples were analyzed by Biotoxtech Co. Ltd. (Cheongju, Korea).

## 5. Conclusions

Overall, our in vitro and in vivo results propose that **LCB01-0699**, a prodrug form of **LCB01-0648**, could be a promising compound for treatment of skin infectious disease caused by multi-drug resistant Gram-positive pathogens.

## Figures and Tables

**Table 1:** Pharmacokinetic parameters of **LCB01-0699** and metabolites in rat plasma after a single 15 mg/kg intravenous (i.v.) injection of **LCB01-0699** (*n* = 3).

Compounds	Parameters	LCB01-0699 (15 mg/kg)
**LCB01-0699**	T_max_ (h) ^a^	0.03 ± 0.00
	C_max_ (mg/L) ^b^	42.9 ± 4.21
	AUC_last_ (mg·h/L) ^c^	5.08 ± 0.68
**LCB01-0648**	T_max_ (h)	0.07 ± 0.03
	C_max_ (mg/L)	41.1 ± 8.09
	AUC_last_ (mg·h/L)	85.0 ± 46.4
**M1**	T_max_ (h)	1.00 ± 0.00
	C_max_ (mg/L)	2.51 ± 0.98
	AUC_last_ (mg·h/L)	9.13 ± 4.23
**M2**	T_max_ (h)	4.00 ± 0.00
	C_max_ (mg/L)	0.72 ± 0.27
	AUC_last_ (mg·h/L)	8.45 ± 2.42
**Sum**	C_max_ (mg/L)	87.2
	AUC_last_ (mg·h/L)	107.7

^a^ T_max_: Time required to reach maximum; ^b^ C_max_: peak concentration; ^c^ AUC_last_: Area under the concentration-time curve from time zero to the last sampling time

**Table 2:** In vivo activity of **LCB01-0699** against *S. aureus* in a mouse model of systemic infection.

Microorganism Inoculum ^a^ (CFU/Mouse) ^b^	Antimicrobial Agent ^c^	MIC ^d^ (mg/L)	ED_50_ (mg/kg) ^e^ (95% Confidence Limits)	
			p.o. ^f^	s.c. ^g^
*S. aureus* Giorgio (methicillin-susceptible *S. aureus*) (5 × 10^7^)				
	**LCB01-0699**	0.5	6.20 (3.58~10.65)	5.51 (3.02~16.91)
	**Linezolid**	2	7.07 (4.07~12.29)	6.26 (3.55~13.89))
*S. aureus* P125 (methicillin-resistant *S. aureus*) (5 × 10^8^)				
	**LCB01-0699**	0.5	2.23 (0.94~5.28)	2.73 (0.90~8.25)
	**Linezolid**	2	7.07 (4.07~12.29)	5.51 (3.02~16.9)

^a^ Bacterial strains were suspended in 0.9% saline solution containing 5% mucin solution; ^b^ CFU: colony-forming units. ^c^ Antibiotics at various dose regimens were administered subcutaneously at 1 and 4 h after the bacterial infection; ^d^ MIC: Minimum inhibitory concentration; ^e^ ED_50_: median effective dose needed to protect 50% of the mice; ^f^ p.o.: orally administered; ^g^ s.c.: the subcutaneous route.
